# Essays on the Effects Produced by Various Processes on Atmospheric Air; with a Particular View to an Investigation of the Acids

**Published:** 1784

**Authors:** 


VII. *Essays on the Effects produced by various processes on atmospheric air; with a particular view to an investigation of the acids. By* M. Lavoisier, *member of the Royal Academy of Sciences at Paris, &c. Translated from the French, by* Thomas Henry, F. R. S. *member of the Medical Society of London, and of the Literary and Philosophical Society of Manchester.* 8vo. Johnson, *London*, 1783. 143 pages.

THESE essays having been originally printed in the Memoirs of the academy of sciences at Paris, have already been noticed in our Journal (*Vols.* II. and IV.). The subjects on which they treat are, the Respiration of animals —Combustion of candles in atmospheric air and in dephlogisticated air—Combustion of Kunckel's phosphorus—Existence of air in the nitrous acid, and the means of decomposing and recomposing that acid—Solution of mercury in vitriolic acid—Combustion of alum with phlogistic substances, and the changes effected on air in which pyrophorus has burned—Vitriolisation of martial pyrites—Nature of the acids, and the principles of which they are composed—Combination of the matter of fire with evaporable rable fluids; and the formation of elaſtic aëriform fluids.——The public are much indebted to Mr. Henry for his accurate tranſlation of theſe ingenious papers, and for a very ſenſible preface, in which, among other things, he gives us ſome intereſting obſervations on phlogiſton.

